# *N* ^2′^,*N* ^5′^-Diisopropylidene­pyrazine-2,5-dicarbohydrazide dihydrate

**DOI:** 10.1107/S1600536808002973

**Published:** 2008-03-07

**Authors:** Miao Ding, Wen-Shi Wu, Hai-Ping Li

**Affiliations:** a The Key Laboratory for Functional Materials of Fujian Higher Education, College of Materials Science and Engineering, Huaqiao University, Quanzhou 362021, Fujian, People’s Republic of China

## Abstract

In the title compound, C_12_H_16_N_6_O_2_·2H_2_O, the organic mol­ecule, except for the methyl H atoms, is essentially planar, the r.m.s. deviation from planarity being 0.044 Å. The crystal structure is stabilized by inter­molecular O—H⋯O and O—H⋯N hydrogen bonds which form chains.

## Related literature

For related literature, see: Wu *et al.* (2003 ▶); Wardell *et al.* (2006 ▶).
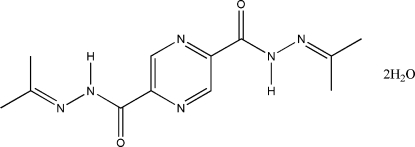


## Experimental

### 

#### Crystal data

C_12_H_16_N_6_O_2_·2H_2_O
*M* *_r_* = 312.34
Triclinic, 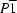

*a* = 7.1924 (5) Å
*b* = 9.9409 (8) Å
*c* = 11.0903 (9) Å
α = 80.261 (6)°
β = 84.605 (5)°
γ = 89.537 (6)°
*V* = 778.03 (10) Å^3^
*Z* = 2
Mo *K*α radiation
μ = 0.10 mm^−1^
*T* = 296 (2) K
0.50 × 0.16 × 0.16 mm

#### Data collection

Bruker P4 diffractometer
Absorption correction: multi-scan (*CrystalClear*; Rigaku, 2000 ▶) *T*_min_ = 0.965, *T*_max_ = 0.984
12989 measured reflections
3643 independent reflections
1540 reflections with *I* > 2σ(*I*)
*R*_int_ = 0.049

#### Refinement

*R*[*F* ^2^ > 2σ(*F* ^2^)] = 0.065
*wR*(*F* ^2^) = 0.209
*S* = 1.00
3643 reflections
224 parameters
H atoms treated by a mixture of independent and constrained refinement
Δρ_max_ = 0.23 e Å^−3^
Δρ_min_ = −0.25 e Å^−3^

### 

Data collection: *CrystalClear* (Rigaku, 2000 ▶); cell refinement: *CrystalClear*; data reduction: *CrystalClear*; program(s) used to solve structure: *SHELXS97* (Sheldrick, 2008 ▶); program(s) used to refine structure: *SHELXL97* (Sheldrick, 2008 ▶); molecular graphics: *SHELXTL* (Sheldrick, 2008 ▶); software used to prepare material for publication: *SHELXTL*.

## Supplementary Material

Crystal structure: contains datablocks global, I. DOI: 10.1107/S1600536808002973/wn2238sup1.cif

Structure factors: contains datablocks I. DOI: 10.1107/S1600536808002973/wn2238Isup2.hkl

Additional supplementary materials:  crystallographic information; 3D view; checkCIF report

## Figures and Tables

**Table 1:** Hydrogen-bond geometry (Å, °)

*D*—H⋯*A*	*D*—H	H⋯*A*	*D*⋯*A*	*D*—H⋯*A*
N5—H5*A*⋯O4	0.86	2.51	3.369 (4)	177
N3—H3*A*⋯O3	0.86	2.52	3.377 (4)	176
O3—H3*D*⋯N4^i^	0.83 (4)	2.15 (4)	2.977 (3)	177 (3)
O3—H3*D*⋯O1^i^	0.83 (4)	2.57 (3)	3.006 (3)	115 (3)
O3—H3*C*⋯N1	0.76 (5)	2.24 (5)	2.974 (3)	162 (5)
O4—H4*C*⋯N2	0.99 (7)	2.05 (7)	2.972 (3)	153 (6)
O4—H4*D*⋯N6^ii^	0.76 (4)	2.25 (4)	3.010 (4)	177 (4)
O4—H4*D*⋯O2^ii^	0.76 (4)	2.58 (4)	2.984 (3)	115 (3)

Symmetry codes: (i) 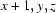; (ii) 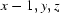.

**Fig. 1.**
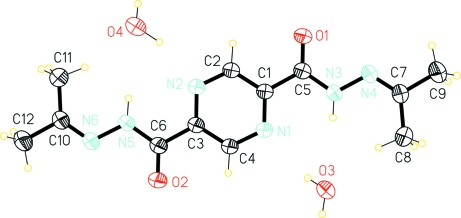
The structure of the title compound. Displacement ellipsoids are drawn at the 30% probability level. Hydrogen atoms are shown as spheres of arbitrary radius.

**Fig. 2.**
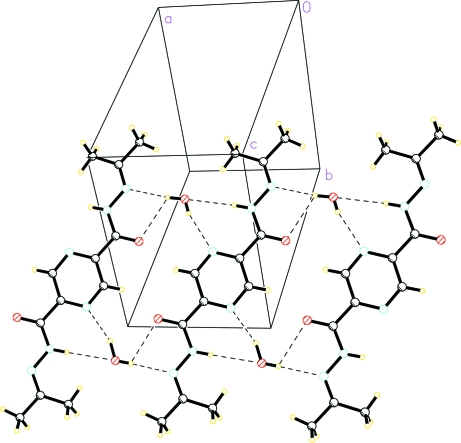
A packing diagram of the title compound, showing the hydrogen bond interactions as dashed lines.

## supplementary crystallographic information

### Comment

Research into amine and hydrazine derivatives has become a major growth area of biological chemistry, structural chemistry, medicine and catalysis. As part of our work in this area, we here present the crystal structure of the title compound.

The structure of the title compound is illustrated in Fig. 1. Except for the H atoms of the methyl groups, the organic molecule is essentially planar, the r.m.s. deviation being 0.044 Å. In the hydrazino fragment, the N—N bond lengths are normal, while the C—N distances are slightly longer than those in pyrazine-2-carbohydrazide (Wardell *et al.*, 2006). In the crystal structure, molecules are linked to form chains by intermolecular O—H···O and O—H···N hydrogen bonds (Fig. 2 and Table 1).

### Experimental

The title compound was prepared by the hydroponics method. 2,5-Pyrazinedihydrazide, as a yellow powder, was synthesized from 2,5-pyrazinedicarboxylic acid dihydrate by esterification and acylation (Wu *et al.*, 2003). 2,5-Pyrazinedihydrazide (20 mg) was dissolved in acetone (20 ml) with stirring for one hour. Transparent orange crystals of the title compound were obtained from the mother liquor by slow evaporation at room temperature after one week.

### Refinement

The positions of the O-bound H atoms were located from a difference Fourier map and refined freely; the refined O—H distances lie in the range 0.76 (4) - 0.99 (7) Å. The H atoms of methyl groups C11 and C12 were also located in a difference map. Other H atoms bonded to C or N were placed in calculated positions. All C– and N-bound C atoms were refined as riding, with N—H = 0.86 Å, C*sp*^2^—H = 0.93 Å and C*sp*^3^—H = 0.96 Å; *U*_iso_(H) = 1.2*U*_eq_(C,*N*).

### Crystal data

**Table:**

C_12_H_16_N_6_O_2_·2H_2_O	*Z* = 2
*M**_r_* = 312.34	*F*_000_ = 332
Triclinic, *P*1	*D*_x_ = 1.333 Mg m^−^^3^
Hall symbol: -P 1	Mo *K*α radiation λ = 0.71073 Å
*a* = 7.1924 (5) Å	Cell parameters from 1817 reflections
*b* = 9.9409 (8) Å	θ = 2.8–22.6º
*c* = 11.0903 (9) Å	µ = 0.10 mm^−^^1^
α = 80.261 (6)º	*T* = 296 (2) K
β = 84.605 (5)º	Prism, orange
γ = 89.537 (6)º	0.50 × 0.16 × 0.16 mm
*V* = 778.03 (10) Å^3^	

### Data collection

**Table:**

Bruker P4 diffractometer	3643 independent reflections
Radiation source: fine-focus sealed tube	1540 reflections with *I* > 2σ(*I*)
Monochromator: graphite	*R*_int_ = 0.049
Detector resolution: 14.6306 pixels mm^-1^	θ_max_ = 27.9º
*T* = 296(2) K	θ_min_ = 1.9º
CCD_Profile_fitting scans	*h* = −9→9
Absorption correction: multi-scan(CrystalClear (Rigaku, 2000)	*k* = −13→13
*T*_min_ = 0.965, *T*_max_ = 0.984	*l* = −14→14
12989 measured reflections	

### Refinement

**Table:**

Refinement on *F*^2^	Secondary atom site location: difference Fourier map
Least-squares matrix: full	Hydrogen site location: inferred from neighbouring sites
*R*[*F*^2^ > 2σ(*F*^2^)] = 0.065	H atoms treated by a mixture of independent and constrained refinement
*wR*(*F*^2^) = 0.209	*w* = 1/[σ^2^(*F*_o_^2^) + (0.0979*P*)^2^ + 0.03*P*] where *P* = (*F*_o_^2^ + 2*F*_c_^2^)/3
*S* = 1.00	(Δ/σ)_max_ < 0.001
3643 reflections	Δρ_max_ = 0.23 e Å^−^^3^
224 parameters	Δρ_min_ = −0.25 e Å^−^^3^
Primary atom site location: structure-invariant direct methods	Extinction correction: none

### Special details

**Table:**

Geometry. All e.s.d.'s (except the e.s.d. in the dihedral angle between two l.s. planes) are estimated using the full covariance matrix. The cell e.s.d.'s are taken into account individually in the estimation of e.s.d.'s in distances, angles and torsion angles; correlations between e.s.d.'s in cell parameters are only used when they are defined by crystal symmetry. An approximate (isotropic) treatment of cell e.s.d.'s is used for estimating e.s.d.'s involving l.s. planes.
Refinement. Refinement of *F*^2^ against ALL reflections. The weighted *R*-factor *wR* and goodness of fit *S* are based on *F*^2^, conventional *R*-factors *R* are based on *F*, with *F* set to zero for negative *F*^2^. The threshold expression of *F*^2^ > σ(*F*^2^) is used only for calculating *R*-factors(gt) *etc*. and is not relevant to the choice of reflections for refinement. *R*-factors based on *F*^2^ are statistically about twice as large as those based on *F*, and *R*- factors based on ALL data will be even larger.

### Fractional atomic coordinates and isotropic or equivalent isotropic displacement parameters (Å^2^)

**Table:**

	*x*	*y*	*z*	*U*_iso_*/*U*_eq_	
N5	0.4457 (3)	0.9409 (2)	0.18538 (19)	0.0570 (6)	
H5A	0.3273	0.9549	0.1898	0.068*	
N2	0.2140 (3)	0.8126 (2)	0.05799 (19)	0.0543 (6)	
N1	0.3605 (3)	0.6324 (2)	−0.09296 (18)	0.0538 (6)	
N3	0.1252 (3)	0.5072 (2)	−0.22285 (19)	0.0563 (6)	
H3A	0.2443	0.4978	−0.2334	0.068*	
N4	0.0048 (3)	0.4399 (2)	−0.2847 (2)	0.0580 (6)	
O1	−0.1200 (2)	0.6055 (2)	−0.12989 (18)	0.0732 (6)	
C3	0.3958 (3)	0.7863 (2)	0.0460 (2)	0.0482 (6)	
C1	0.1776 (3)	0.6578 (2)	−0.0798 (2)	0.0463 (6)	
N6	0.5616 (3)	1.0070 (2)	0.25097 (19)	0.0580 (6)	
C5	0.0469 (4)	0.5873 (3)	−0.1459 (2)	0.0515 (6)	
C6	0.5253 (4)	0.8541 (3)	0.1146 (2)	0.0578 (7)	
O2	0.6916 (3)	0.8306 (2)	0.1056 (2)	0.0898 (8)	
C2	0.1058 (3)	0.7480 (3)	−0.0051 (2)	0.0543 (7)	
H2A	−0.0220	0.7639	0.0012	0.065*	
C4	0.4690 (3)	0.6970 (3)	−0.0294 (2)	0.0551 (7)	
H4A	0.5970	0.6818	−0.0359	0.066*	
C10	0.4878 (4)	1.0898 (3)	0.3168 (2)	0.0573 (7)	
C12	0.6179 (5)	1.1587 (4)	0.3842 (3)	0.0696 (9)	
H12A	0.592 (4)	1.136 (3)	0.468 (3)	0.084*	
H12B	0.609 (4)	1.264 (3)	0.364 (2)	0.084*	
H12C	0.742 (5)	1.131 (3)	0.363 (3)	0.084*	
C7	0.0747 (4)	0.3704 (3)	−0.3630 (2)	0.0581 (7)	
C9	−0.0602 (4)	0.2998 (3)	−0.4275 (3)	0.0764 (9)	
H9A	−0.1853	0.3262	−0.4041	0.115*	
H9B	−0.0484	0.2028	−0.4048	0.115*	
H9C	−0.0329	0.3253	−0.5147	0.115*	
C11	0.2879 (4)	1.1256 (3)	0.3329 (3)	0.0796 (9)	
H11A	0.2339	1.0799	0.4115	0.095*	
H11B	0.2237	1.0985	0.2688	0.095*	
H11C	0.2754	1.2226	0.3280	0.095*	
C8	0.2769 (4)	0.3527 (3)	−0.4004 (3)	0.0831 (10)	
H8A	0.2988	0.3727	−0.4884	0.125*	
H8B	0.3122	0.2603	−0.3720	0.125*	
H8C	0.3498	0.4138	−0.3651	0.125*	
O3	0.5953 (4)	0.4902 (3)	−0.2671 (2)	0.0869 (8)	
O4	−0.0209 (4)	0.9821 (3)	0.2080 (3)	0.0965 (9)	
H3D	0.710 (6)	0.479 (3)	−0.271 (3)	0.106 (14)*	
H3C	0.556 (7)	0.526 (5)	−0.215 (4)	0.15 (2)*	
H4C	0.032 (9)	0.939 (7)	0.138 (6)	0.28 (3)*	
H4D	−0.127 (6)	0.985 (4)	0.219 (3)	0.096 (14)*	

### Atomic displacement parameters (Å^2^)

**Table:**

	*U*^11^	*U*^22^	*U*^33^	*U*^12^	*U*^13^	*U*^23^
N5	0.0391 (12)	0.0708 (14)	0.0701 (14)	0.0022 (10)	−0.0142 (10)	−0.0330 (12)
N2	0.0372 (12)	0.0665 (14)	0.0653 (13)	0.0044 (10)	−0.0077 (10)	−0.0272 (11)
N1	0.0385 (12)	0.0677 (14)	0.0604 (13)	0.0037 (10)	−0.0050 (10)	−0.0260 (11)
N3	0.0395 (12)	0.0720 (14)	0.0655 (13)	−0.0035 (11)	−0.0071 (10)	−0.0333 (11)
N4	0.0466 (13)	0.0693 (14)	0.0660 (14)	−0.0023 (11)	−0.0126 (10)	−0.0298 (12)
O1	0.0349 (11)	0.0945 (15)	0.1046 (15)	0.0051 (10)	−0.0114 (9)	−0.0553 (12)
C3	0.0375 (14)	0.0582 (15)	0.0520 (14)	0.0020 (12)	−0.0073 (11)	−0.0166 (12)
C1	0.0358 (14)	0.0528 (15)	0.0528 (15)	0.0015 (11)	−0.0047 (11)	−0.0159 (12)
N6	0.0449 (13)	0.0728 (15)	0.0641 (13)	−0.0030 (11)	−0.0125 (10)	−0.0293 (12)
C5	0.0415 (15)	0.0583 (16)	0.0574 (15)	0.0009 (13)	−0.0066 (12)	−0.0167 (13)
C6	0.0413 (16)	0.0764 (18)	0.0618 (17)	0.0017 (14)	−0.0084 (13)	−0.0274 (14)
O2	0.0381 (11)	0.1373 (19)	0.1155 (17)	0.0089 (11)	−0.0134 (10)	−0.0795 (15)
C2	0.0362 (14)	0.0670 (17)	0.0659 (16)	0.0045 (12)	−0.0075 (12)	−0.0278 (14)
C4	0.0363 (14)	0.0734 (18)	0.0621 (16)	0.0030 (13)	−0.0061 (11)	−0.0297 (14)
C10	0.0491 (17)	0.0667 (17)	0.0614 (16)	−0.0036 (14)	−0.0110 (13)	−0.0225 (14)
C12	0.0596 (19)	0.085 (2)	0.0721 (19)	−0.0070 (18)	−0.0121 (16)	−0.0323 (18)
C7	0.0552 (17)	0.0645 (17)	0.0602 (16)	−0.0022 (14)	−0.0103 (13)	−0.0239 (14)
C9	0.068 (2)	0.086 (2)	0.088 (2)	−0.0015 (17)	−0.0230 (16)	−0.0406 (17)
C11	0.0578 (19)	0.091 (2)	0.104 (2)	0.0091 (16)	−0.0141 (16)	−0.0544 (19)
C8	0.061 (2)	0.114 (3)	0.090 (2)	0.0072 (18)	−0.0084 (16)	−0.059 (2)
O3	0.0485 (14)	0.128 (2)	0.1033 (18)	0.0056 (13)	−0.0192 (12)	−0.0660 (16)
O4	0.0506 (15)	0.122 (2)	0.137 (2)	0.0060 (14)	−0.0248 (14)	−0.0695 (17)

### Geometric parameters (Å, °)

**Table:**

N5—C6	1.352 (3)	C10—C11	1.480 (4)
N5—N6	1.393 (3)	C10—C12	1.489 (4)
N5—H5A	0.8600	C12—H12A	0.92 (3)
N2—C2	1.330 (3)	C12—H12B	1.03 (3)
N2—C3	1.330 (3)	C12—H12C	0.95 (3)
N1—C4	1.335 (3)	C7—C8	1.492 (4)
N1—C1	1.337 (3)	C7—C9	1.502 (3)
N3—C5	1.346 (3)	C9—H9A	0.9600
N3—N4	1.394 (3)	C9—H9B	0.9600
N3—H3A	0.8600	C9—H9C	0.9600
N4—C7	1.264 (3)	C11—H11A	0.9600
O1—C5	1.213 (3)	C11—H11B	0.9600
C3—C4	1.388 (3)	C11—H11C	0.9600
C3—C6	1.489 (3)	C8—H8A	0.9600
C1—C2	1.386 (3)	C8—H8B	0.9600
C1—C5	1.492 (3)	C8—H8C	0.9600
N6—C10	1.272 (3)	O3—H3D	0.83 (4)
C6—O2	1.215 (3)	O3—H3C	0.76 (5)
C2—H2A	0.9300	O4—H4C	0.99 (7)
C4—H4A	0.9300	O4—H4D	0.76 (4)
			
C6—N5—N6	117.9 (2)	C11—C10—C12	116.8 (2)
C6—N5—H5A	121.1	C10—C12—H12A	112.0 (18)
N6—N5—H5A	121.1	C10—C12—H12B	112.1 (16)
C2—N2—C3	116.6 (2)	H12A—C12—H12B	106 (2)
C4—N1—C1	116.5 (2)	C10—C12—H12C	108.8 (17)
C5—N3—N4	117.1 (2)	H12A—C12—H12C	108 (3)
C5—N3—H3A	121.5	H12B—C12—H12C	110 (2)
N4—N3—H3A	121.5	N4—C7—C8	127.2 (2)
C7—N4—N3	118.5 (2)	N4—C7—C9	116.7 (3)
N2—C3—C4	121.7 (2)	C8—C7—C9	116.1 (2)
N2—C3—C6	119.6 (2)	C7—C9—H9A	109.5
C4—C3—C6	118.7 (2)	C7—C9—H9B	109.5
N1—C1—C2	121.4 (2)	H9A—C9—H9B	109.5
N1—C1—C5	119.6 (2)	C7—C9—H9C	109.5
C2—C1—C5	119.0 (2)	H9A—C9—H9C	109.5
C10—N6—N5	118.2 (2)	H9B—C9—H9C	109.5
O1—C5—N3	123.8 (2)	C10—C11—H11A	109.4
O1—C5—C1	119.8 (2)	C10—C11—H11B	109.8
N3—C5—C1	116.4 (2)	H11A—C11—H11B	109.8
O2—C6—N5	123.5 (2)	C10—C11—H11C	109.9
O2—C6—C3	120.6 (2)	H11A—C11—H11C	109.8
N5—C6—C3	115.9 (2)	H11B—C11—H11C	108.2
N2—C2—C1	122.1 (2)	C7—C8—H8A	109.5
N2—C2—H2A	118.9	C7—C8—H8B	109.5
C1—C2—H2A	118.9	H8A—C8—H8B	109.5
N1—C4—C3	121.8 (2)	C7—C8—H8C	109.5
N1—C4—H4A	119.1	H8A—C8—H8C	109.5
C3—C4—H4A	119.1	H8B—C8—H8C	109.5
N6—C10—C11	127.2 (2)	H3D—O3—H3C	115 (4)
N6—C10—C12	116.0 (3)	H4C—O4—H4D	117 (4)
			
C5—N3—N4—C7	175.9 (2)	N2—C3—C6—O2	179.5 (3)
C2—N2—C3—C4	0.7 (4)	C4—C3—C6—O2	−0.7 (4)
C2—N2—C3—C6	−179.6 (2)	N2—C3—C6—N5	−0.5 (4)
C4—N1—C1—C2	0.7 (4)	C4—C3—C6—N5	179.3 (2)
C4—N1—C1—C5	−178.8 (2)	C3—N2—C2—C1	−0.1 (4)
C6—N5—N6—C10	179.3 (2)	N1—C1—C2—N2	−0.6 (4)
N4—N3—C5—O1	−1.4 (4)	C5—C1—C2—N2	178.9 (2)
N4—N3—C5—C1	179.63 (19)	C1—N1—C4—C3	−0.2 (4)
N1—C1—C5—O1	176.7 (2)	N2—C3—C4—N1	−0.5 (4)
C2—C1—C5—O1	−2.8 (4)	C6—C3—C4—N1	179.7 (2)
N1—C1—C5—N3	−4.3 (3)	N5—N6—C10—C11	0.0 (4)
C2—C1—C5—N3	176.2 (2)	N5—N6—C10—C12	−179.5 (2)
N6—N5—C6—O2	0.2 (4)	N3—N4—C7—C8	−1.3 (4)
N6—N5—C6—C3	−179.8 (2)	N3—N4—C7—C9	−180.0 (2)

### Hydrogen-bond geometry (Å, °)

**Table:**

*D*—H···*A*	*D*—H	H···*A*	*D*···*A*	*D*—H···*A*
N5—H5A···O4	0.86	2.51	3.369 (4)	177
N3—H3A···O3	0.86	2.52	3.377 (4)	176
O3—H3D···N4^i^	0.83 (4)	2.15 (4)	2.977 (3)	177 (3)
O3—H3D···O1^i^	0.83 (4)	2.57 (3)	3.006 (3)	115 (3)
O3—H3C···N1	0.76 (5)	2.24 (5)	2.974 (3)	162 (5)
O4—H4C···N2	0.99 (7)	2.05 (7)	2.972 (3)	153 (6)
O4—H4D···N6^ii^	0.76 (4)	2.25 (4)	3.010 (4)	177 (4)
O4—H4D···O2^ii^	0.76 (4)	2.58 (4)	2.984 (3)	115 (3)

Symmetry codes: (i) *x*+1, *y*, *z*; (ii) *x*−1, *y*, *z*.
